# Complexes of Pd(II) and Pt(II) with 9-Aminoacridine: Reactions with DNA and Study of Their Antiproliferative Activity

**DOI:** 10.1155/2007/98732

**Published:** 2007-08-29

**Authors:** X. Riera, V. Moreno, C. J. Ciudad, V. Noe, M. Font-Bardía, X. Solans

**Affiliations:** ^1^Departamento de Química Inorgànica, Facultat de Química, Universitat de Barcelona, Martí i Franquès 1-11, 08028 Barcelona, Spain; ^2^Departamento de Bioquímica i Biologia Molecular, Facultat de Biología, Universitat de Barcelona, avenue Diagonal 645, 08028 Barcelona, Spain; ^3^Departamento de Cristal.lografia, Mineralogia i Dipòsits Minerals, Facultat de Geología, Universitat de Barcelona, C/ Martí i Franquès s/n, 08028 Barcelona, Spain

## Abstract

Four new metal complexes {M = Pd(II) or Pt(II)} containing the ligand 9-aminoacridine (9AA) were prepared. The compounds were characterized by FT-IR and ^1^H, ^13^C, and ^195^Pt NMR spectroscopies. Crystal structure of the palladium complex of formulae [Pd(9AA)(*μ*-Cl)]_2_ · 2DMF was determined by X-ray diffraction. Two 9-acridine molecules in the imine form bind symmetrically to the metal ions in a bidentate fashion through the imine nitrogen atom and the C(1) atom of the aminoacridine closing a new five-membered ring. By reaction with phosphine or pyridine, the Cl bridges broke and compounds with general formulae [Pd(9AA)Cl(L)] (where L = PPh_3_ or py) were formed. A mononuclear complex of platinum of formulae [Pt(9AA)Cl(DMSO)] was also obtained by direct reaction of 9-aminoacridine and the complex [PtCl_2_(DMSO_2_]. The capacity of the compounds to modify the secondary and tertiary structures of DNA was evaluated by means of circular dichroism and electrophoretic mobility. Both palladium and platinum compounds proved active in the modification of both the secondary and tertiary DNA structures. AFM images showed noticeable modifications of the morphology of the plasmid pBR322 DNA by the compounds probably due to the intercalation of the complexes between base pairs of the DNA molecule. Finally, the palladium complex was tested for antiproliferative activity against three different human tumor cell lines. The results suggest that the palladium complex of formula [Pd(9AA)(*μ*-Cl)]_2_ has significant antiproliferative activity, although it is less active than cisplatin.

## 1. INTRODUCTION

Intercalation between parallel base pairs is frequently one of the possible modes of interaction of DNA molecule with active drugs. Classic intercalators are plane aromatic molecules such as acridines, phenantrolines, or phorphyrins. The family of the aminoacridines has been extensively studied [1].

The applications in medicine of these chemical compounds started in early 20th century, when derivatives of *crisaniline* were found to be active against malaria, and *euflavine* and *proflavine* were used as antibacterial agents. These compounds were replaced by *aminacrine* (9-aminoacridine) which has similar effects. Afterwards, when the affinity of the acridines for the nucleic acids and their intercalator effects were established [2, 3], the research focused on their possible applications as anticancer agents. However, tests conducted with simple acridines had very low activity as antitumor drugs [4]. Systematic studies of the relationship between the antitumor activity and several factors such as the lipophilicity-hydrophilicity of the derivatives, their electronic and steric effects have also been carried out [5]. Recently, 9-aminoacridine has been assayed, in comparison with other acridine derivatives such as pharmacotherapeutic for prion disease Creutzfeldt-Jacob disease (CJD) [6]. The incorporation of bulky groups into the acridine moiety does not allow the intercalation of the chromophor between the DNA base pairs and causes decreasing of the antitumor activity [7].

In parallel, research focussed on the design of compounds containing several acridine molecules joined by carbon chains in order to create a DNA polyintercalator effect [8–10] and on the preparation of coordination compounds with one acridine group as a ligand [11, 12] with the objective of obtaining molecules capable of interaction with DNA by intercalation and by metal-base covalent bond. Intercalation is a kinetically labile interaction and the additional fixation of the molecule to DNA by a covalent bond via the metal atom can potentially increase the activity of these systems as antitumor agents.

**Scheme 1:**
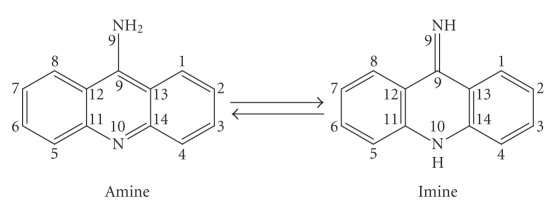
Schematic representation of the two tautomeric forms of the 9-aminoacridine.

The 9-aminoacridine (9AA) molecule has two symmetrically distributed nitrogen atoms with nucleophilic properties: the exocyclic N(9) and the endocyclic N(10), which can coordinate to transition metal atoms. Two tautomeric forms of the free 9-aminoacridine moiety can be adopted in solution (see scheme1).

Rak et al. [13–15] have described the presence of these two forms in solution and have demonstrated that the equilibrium composition changes when the solvent and temperature change, although the authors do not conclude about what isomer is preferentially formed.

An additional interest of this ligand resides in the fact that the DNA interaction occurs by intercalation of 9-aminoacridine between the base pairs of the biomolecule and this is, theoritically, compatible with the preferential coordination of platinum to DNA [16–20] by the nitrogen N(7) of the purine bases on the major groove.

Sundquist et al. [21] have described the synthesis of coordination compounds of formulae *cis*-[PtCl(9AA)-(NH_3_)_2_](NO_3_) and *cis*-[Pt(9AA)_2_ (NH_3_)_2_](NO_3_)_2_ prepared reacting a derivative of *cisplatin* with the ligand 9-aminoacridine (9AA). In the two complexes, the ligand is coordinated in its imine form, which binds to the platinum atom in a monodentate fashion by the exocyclic nitrogen atom. Natile and col. [22–24] have investigated the reaction of 9-[(2-aminoethyl)amino]acridines with platinum(II) substrates and also have studied the endocyclic versus exocyclic N-coordination to platinum(II) and the role of metal ions and hydrogen bond acceptors in the tautomeric equilibrium of nitro-derivatives of 9-aminoacridines.

More recently, the interaction of 9-aminoacridine-carboxamide platinum complexes with DNA has been investigated, particulary their DNA sequence specificity and binding kinetics [25]. The presence of the 9-amino substituent produces the effect of shifting away from runs of consecutive guanines (the main binding site for cisplatin). However, an acridinecarboxamide platinum complex showed a similar sequence specifity to cisplatin. The same authors prepared *cis*-dichloroplatinum(II) complexes tethered to 9-aminoacridine-4-carboxamides and assessed their activity in several resistant cell lines in vitro [26]. The sequence specifity and kinetics of DNA adduct formation for the aforementioned compounds with HeLa cells were also compared with those of cisplatin, resulting 4-fold faster for DNA-targeted Pt complexes [27]. Platinum-acridine conjugates were also prepared and tested against several tumor cell lines resulting active at micromolar concentrations. Mono- and bis-acridinylthiourea platinum (II) complexes were synthesized by Bierbach's research group [28–30] with the aim of studying DNA strands cleavage and binding modes. The interaction of ACRAMTU-Pt complexes {ACRAMTU = 1-[2-(acridin-9-ylamino)ethyl]-1,3-dimethylthiourea} with DNA have been extensively studied by Bierbach et al. [31–36]. Recent results from their biophysical and biochemical studies suggest interesting binding mechanisms to the DNA molecule.

Finally, metal derivatives of 9-aminoacridine have been synthesized as precursors for radio iodination for potential use in radionuclide therapy [37].

Here, the synthesis of new Pd(II) and Pt(II) complexes of the classic intercalator 9-aminoacridine (9AA), where the ligand acts as a (C,N)^− ^bidentate group, is presented. The study of their chemical, structural properties and reactivity with DNA, as well as their antiproliferative behavior with selected tumor cell lines are also described.

## 2. EXPERIMENTAL

### 2.1 Materials and methods

The complexes were prepared using K_2_[PdCl_4_] and K_2_[PtCl_4_] from Johnson Matthey (Reading, UK); the solvents used were purchased from Fluka (Madrid, Spain); and 9-aminoacridine, *Calf Thymus*-DNA, and EDTA from Sigma-Aldrich (Madrid, Spain).

Elemental analyses were carried out on a Carlo Erba 1500 microanalyzer at the Serveis Cientèfico-Tècnics of the University of Barcelona. Chlorine in the compounds was analyzed by the Shöniger method and potentiometric titration in a titroprocessor Metrohm 636 provided with a silver combined electrode. Platinum was determined by flameless atomic absorption spectroscopy (FAAS) using a Unicam 939 AA spectrometer with graphite furnace; the measurements were done by addition and the sample was dissolved in DMF to a concentration of 0.3 mM. Palladium was measured by inductively coupled plasma-optical emission spectroscopy (ICP-OES) using a Thermo Jarrell Ash polyscan GIE in standard conditions at a wavelength of 340–458 nm. The samples required prior mineralization to ICP after which they were digested with concentrated nitric and perchloric acids in pyrex tubes at 220 °C; palladium was dissolved in 100 ml of Milli-Q water in acid medium [38]. The IR spectra were recorded in a solid state (KBr pellets) on an FT-IR Nicolet 5DZ spectrometer in the 4000–400cm^−1^ range and on an FT-IR Bomen DA-3 (CsBr pellets) for the 400–200 cm^−1^ range. ^1^H{^13^C}, ^13^C{^1^H}, and ^195^Pt{^1^H} NMR spectra were recorded on a Bruker DRX 250 spectrometer using CDCl_3_ as solvent in the case of ^1^H and ^13^C spectra and DMSO-*d*_6_ in the case of ^195^Pt spectrum due to the low solubility in CDCl_3_ of the platinum complex. Chemical shifts were measured relative to TMS in the case of ^1^H and ^13^C, and to K_2_PtCl_6_ in the case of ^195^Pt NMR spectra. Mass spectra were run on a Fisons VG Quattro triple quadrupole analyzer in the 1800–200 m/z range using MeCN as solvent under electrospray (ESP-MS).

### 2.2 Syntheses of the complexes

**[Pd(9AA)(*μ*-Cl)]_2_ ⋅ 2DMF**

A solution of 0.192 g (0.50 mM) of *cis*-[PdCl_2_(PhCN)_2_] [39] in 10 mL of CHCl_3_ was added to 40 mL of a solution of 0.097 g (0.50 mM) of 9-aminoacridine in CHCl_3_. The mixture was refluxed at 60 °C for 12 hours. A brown solid was formed after cooling. The resulting precipitate was washed with ethanol and ethylic ether, and dried overnight under silica gel. The solid was dissolved in a minimum amount (ca. 5 mL) of a mixture of CHCl_3_:DMF (100:50) and the solution was eluted in a SiO_2_ column (30 × 2 cm) using CHCl_3_:DMF (100:50) as eluent. The orange band collected from the SiO_2_ column was concentrated by evaporation under vacuum and a brownish solid was obtained. Adequate crystals for X-ray diffraction were obtained from a fraction collected from the column after slow evaporation of the solvent Yield: 35%. [Pd_2_C_32_H_32_N_6_CI_2_O_2_] requires: C, 47.07; N, 10.29; H, 3.92; found: C, 46.75; N, 10.40; H, 4.20.

**[Pd(9AA)Cl(py- d_5_)]**

This compound was obtained at NMR scale. 0.008 g (0.0098 mM) of [Pd(9AA)( *μ*-Cl)]_2_ ⋅ 2DMF were dissolved in 0.7 mL of DMSO-*d*_6_ and 2 drops of pyridine-*d*_5_. The solution immediately changed its color from brownish to strong yellow.

**[PdCl(9AA)(PPh_3_)]**

In a first step, this compound was prepared at NMR scale, but further it was also isolated in a solid state. 0.004 g (0.0049 mM) of [Pd(*μ*-Cl)(9AA)]_2_ ⋅ 2DMF were dissolved in 0.4 mL of DMSO-*d*_6_ and 0.0026 g (0.0098 mM) of triphenylphosphine dissolved in 0.3 mL of DMSO-*d*_6_ were added. The solution changed immediately from brown color to strong yellow.

The product in solid state was prepared by the following procedure: 0.0257 g (0.0098 mM) of triphenyl/phosphine, dissolved in the minimum amount of acetone were added to a suspension of 0.04 g (0.049 mM) of [Pd(*μ*-Cl)(9AA)]_2_ ⋅ 2DMF in 30 mL of acetone. The mixture was stirred at room temperature for 1 hour until the solid disappeared. The final yellow solution was concentrated in a rotavapor and an oil was obtained. 20 mL of diethylether were added to the oil and a yellow precipitate was formed which was filtered and dried. Yield: 82%. [PdC_31_H_24_N_2_C1P] requires: C, 62.32; N, 4.69; H, 4.02; found: C, 62.80; N, 4.50; H, 4.20.

**[PtCl(9AA)(DMSO)]**

A suspension of 0.106 g (0.25 mM) of cis-[PtCl_2_(DMSO)_2_] and 20 mL of methanol was refluxed until the solid dissapeared. A solution of 0.097 g (0.50 mM) of 9-aminoacridine in the minimum amount of methanol was added and the resultant mixture was refluxed for 16 hour. When the solvent was eliminated, a brownish solid remained in the bottle. The solid was dissolved in 20mL of acetone. The solution was filtered on Ceolite, and finally, n-hexane was added until precipitation of a yellow solid, which was filtered, washed with small amounts of n-hexane, and dried at the air. Yield: 51%. PtC_15_H_15_N_2_ClOS requires: C, 35.86; N, 5.58; H, 2.99; S, 6.37; found: C, 36.10; N, 5,40; H, 3.10; S, 6.40.

### 2.3 X-ray diffraction

A [Pd(9AA)(*μ*-Cl)]_2_ ⋅ 2DMF prismatic crystal (0.1 × 0.1 × 0.2 mm) was selected and mounted on an Enraf-Nonius CAD4 four-circle diffractometer. Unit-cell parameters were determined from automatic centering of 25 reflections (12 < Θ < 21°) and refined by least-squares method. Intensities were collected with graphite monochromatized MoK *α* radiation, using w/2Θ scan technique. 4911 reflections were measured in the range 2.60 > Θ > 29.97. 4715 of which were non-equivalent by symmetry (R_int_ (on I) = 0.018). 3681 reflections were assumed as observed applying the condition I > 2*σ* (I). Three reflections were measured every two hours as orientation and intensity control; significant intensity decay was not observed. Lorentz-polarization, but not absorption corrections, was made.

The structure was solved by direct methods using SHELXS computer program [40] for determination of crystal structures and refined by full-matrix least-squares method with SHELX93 computer program [41] using 4665 reflections, (very negative intensities were not assumed). The function minimized was Σw [(Fo)^2^ − (Fc)^2^]^2^, where w = [*σ* ^2^(I) + (0.0683 P)^2^]^−1^, and P = [(Fo)^2^ + 2 (Fc)^2^]/3; f, f′, and f″ were taken from the international tables of X-Ray crystallography [42]. All H atoms were computed and refined with an overall isotropic temperature factor, using a riding model. Hydrogen coordinates as well as anisotropic thermal parameters are included as supplementary material.

### 2.4 Formation of drug-DNA complexes

Stock solutions of each compound (1mg/mL) were stored in the dark at room temperature until used. Drug-DNA complex formation was accomplished by addition of CT DNA (*Calf thymus* DNA) to aliquots of each of the compounds at different concentrations in TE buffer (50 mM NaCl, 10 mM Tris-HCl, 0.1 mM EDTA, pH = 7.4). The amount of compound added to the DNA solution was designated as (r_i_) (the input molar ratio of Pt, Pd, or 9-aminoacridine to nucleotide). The mixture was incubated at 37°C for 24 hours.

### 2.5 Circular Dichroism

The CD spectra of the complex-DNA compounds (DNA concentration 20 mg/mL, (r_i_) = 0.05, 0.10, 0.30, and 0.50) were recorded at room temperature on a JASCO J720 spectropolarimeter with a 450 W xenon lamp using a computer for spectral subtraction and noise reduction. Each sample was scanned twice in a range of wavelengths between 220–360 nm. The CD spectra drawn are the mean of three independent scans. The data are expressed as mean residue molecular ellipticity [ Θ ] in degree cm ^−1^ ⋅ dmol^−1^.

### 2.6 Determination of Pt and Pd bound to DNA

The drug-DNA complex solutions used for CD experiments were kept; the DNA was afterwards precipitated twice with 2.5 volumes of cold ethanol and 0.1 volume of 3M NaAcO, pH 4.8. The DNA was washed in 70% ethanol and suspended in 1mL of TE buffer. The amount of DNA in each sample was measured by a double-beam Shimazdu UV-2101-PC spectrometer. The platinum and palladium bound to the DNA was determined by Inductively Coupled Plasma-Mass spectrometer (ICP-MASS) Perkin Elmer ELAN-500. The assays were performed in triplicate.

### 2.7 Electrophoretic mobility in agarose gel

Commercial solution of pBR322 plasmid DNA, 0.25 *μ*g/ *μ*L was used for electrophoretic mobility experiments.

4 *μ*L of charge marker were added to aliquot parts of 20 *μ*L of the adducts complex: DNA previously incubated at 37°C for 24 hours. The mixture was electrophoretized in agarose gel (l% in TBE buffer) for 5 hours at 1.5V/cm. Afterwards, the DNA was dyed with thydium bromide solution (0.5 *μ*g/mL en TBE) for 20 minutes.

Samples of DNA and adduct cisplatin: DNA were used as control. The experiment was carried out in an ECOGEN horizontal tank connected to a PHARMACIA GPS 200/400 variable potential power supply.

### 2.8 Atomic force microscopy (TMAFM)

pBR322 DNA was heated at 60° for 10 minutes to obtain OC form. Stock solution is 1mg/mL in a buffer solution of HEPES. Each sample contains 1 *μ*L of DNA pBR322 of concentration 0.25 *μ*g/ *μ*L for a final volume of 50 *μ*L. The amount of drug added was expressed as (r_i_), ratio between the molar concentration of drug to number of base pairs.

Images are obtained with a Nanoscope III multimode AFM of Digital Instruments Inc. operating in tapping mode.

### 2.9 Tumor cell lines and culture conditions

Three different tumor cell lines were used in these experiments: MCF-7 breast cancer cell line, DU-145 prostate cancer cell line, and HeLa cervix cancer cell line. The protocols used in each case were the following: MCF-7 cells were routinely maintained in DMEM medium supplemented with 10% of fetal bovine serum (FBS), DU-145 cells in RPMI medium supplemented with 10% of FBS and 2 mmol/L of glutamine, and HeLa cells in Ham's F-12 medium. The cultures were kept in an incubator at a highly humidified atmosphere of 95% air with 5% CO_2_ at 37 °C.

The cells were collected from the medium and were counted with a hemocytometer. Aliquot parts of 100 *μ*L were placed in 96 wells (2000 or 3000 cells per well for MCF-7 i DU-145, resp.). The cells were preincubated without drug for 48 hours (MCF-7) and for 72 hours (DU-145), at 37 °C and 5% CO_2_ atmosphere with 95% of relative humidity. Immediately before to be used, the complexes were solved in sterile water or DMSO/H_2_O mixture at a stock concentration of 1mg/mL and filtered. Aliquot parts of these solutions were added to each well (between 20 and 50 *μ*L of compound depending on the final concentration required). In any case, the concentration of DMSO in the solution in contact with the cells was higher than 1%. After addition of the compound, the cells were incubated for 48 hours in the above conditions. 20 *μ*L of MTT solution (5 mg/mL in PBS) were added to each well and were incubated for 3-4 hours more. Then, 150 *μ*L of solution of solubilization of MTT-formazan crystalls formed (500 mL DMF, 200 g SDS, 20 mL glacial acetic acid, 10 mL of HCl 2M and water until 1L) were added. The cellular density was calculated in both, the control cultures and the treated cultures, measuring the absorbance at 570 nm in an ELISA reader Labsystems Multiskan Multisoft. The IC_50_ values were calculated from the graphic representation of cell survival percentage in function of drug (in *μ*M). The data were obtained from four independent experiments.

In the case of the HeLa cells, the methodology followed was basically identical with the following differences: number of cells per well, 2000, preincubation for 24 hours, incubation for 24 hours, and concentrations of 0.1, 1, 10 i 50 *μ*M; solution of solubilization of MTT-formazan crystalls, DMSO; wavelength of reading, 490 nm; reader of microplates ELISA, ELX800G from Bio-Tek Instruments Inc.

## 3. RESULTS AND DISCUSSION

The main objective of this work was the synthesis, characterization, and biological studies of compounds of general formulae *cis*-[MCl_2_(9AA)_2_] (M = Pd ∘ Pt, 9AA = 9-aminoacridine).

One of the most common synthetic routes of complexes *cis*-[MX_2_ (L)] or *cis*-[MX_2_ (L)_2_] consists on the reaction of the ligand (L) with the compounds M^1^_2_ [MCl_4_] (where M^I^ = Na or K, and M = Pd or Pt). The reaction between the 9-aminoacridine and the compounds K_2_[MCl_4_] (M = Pd ∘ Pt) was not successful due to their low solubility in organic solvents. (i.e., methanol, ethanol, CHCl_3_, acetone), in which the ligand is soluble. The use of Na_2_[PdCl_4_] yielded a brown solid insoluble in the usual solvents which could not be completly characterized. The ^1^H NMR and elemental analysis results suggest that the solid is mainly a mixture of the coordination compounds with 1:1 and 1:2 (metal:ligand) stochiometries together with lower concentration unidentified species.

**Scheme 2:**
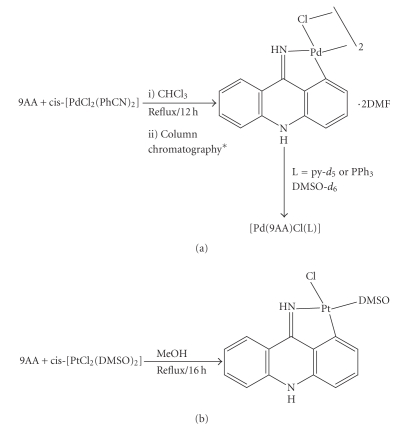
Scheme of the synthesis of the complexes (a) [Pd(9AA)(*μ*-Cl)]_2_ and [Pt(9AA)(L)] (L = py- *d*_5_ or PPh_3_) and (b) [Pt(9AA)Cl(DMSO)]: *De SiO_2_. The eluted used was a mixture CHCl_3_:DMF (100:50).

Using the compound *cis*-[PdCl_2_(PhCN)_2_] as starting material in stronger reaction conditions and after purification of the reaction product by column chromatography, the dimeric cyclopalladate complex bridged through chlorine atoms of formula [Pd(9AA)(*μ*-Cl)]_2_ (see Scheme 2(a)) was isolated.

The cycloplatinated compound of formulae [Pt(9AA)Cl-(DMSO)] (Scheme 2(b)), was synthesized by reaction of 9-aminoacridine and *cis*-[PtCl_2_(DMSO)_2_], 2:1, in methanol reflux. It was not possible to isolate the coordination compound *cis*-[PtCl_2_(L)_2_] in spite of that several reaction times (between 30 minutes and 24 hours) and temperatures (between 25 and 70°C) were assayed. The activation of the C–H bond is clearly favored in the conditions used. To the authors' knowledge, [Pd(9AA)(*μ*-Cl)]_2_ and [Pt(9AA)Cl(DMSO)] are the first metallocycle derivatives of the 9-aminoacridine, where the ligand behaves as a bidentate (C,N)^−^ group. Two cycloplatinated compounds derivatives of 1-nitro-9-[{(2-alkylamino)ethyl}amino]acridine {alkyl = CH_2_-CH_3_ or CH_3_} were previously described by Ceci et al. [22]. In those compounds, the ligand acts as a monoanionic terdentate (C,N,N′)^−^, where the platinum atom binds simultaneously to two exocyclic nitrogen atoms and to the C^8^ carbon, with the fourth coordination site occupied by a chlorine atom.

### 3.1 Reactivity of the “Pd(*μ*-Cl)_2_ Pd” moiety in the dinuclear compound [Pd(9AA)(*μ*-Cl)]_2_

The reaction of the compound [Pd(9AA)(*μ*-Cl)]_2_ with pyridine-*d*_5_ (py-*d*_5_) or with triphenylphosphine (PPh_3_) yielded the corresponding monomeric compounds [Pd(9AA)Cl(L)] (L = py-*d*_5_, PPh_3_) (see Scheme 2(a)). The lability of the Pd–N bond is lower than that described for cyclopalladated compounds of five-membered rings derivatives of *N*-benzylidenaniline [43] which react with triphenylphosphine to give [Pd(C^N)X(L)_2_] after break of the Pd–N bond (Scheme 2(a)). In spite of the addition of excess of pyridine- *d*_5_ or PPh_3_, the substitution of the second chloride ligand was not observed.

The new compounds prepared [Pd(9AA)(*μ*-Cl)]_2_ ⋅ 2DMF, [Pd(9AA)Cl(L)] (L = py- *d*_5_ or PPh_3_) and [Pt(9AA)Cl(DMSO)] were characterized by IR and ^1^H, ^13^C, ^31^P {in the case of [Pd(9AA)Cl(PPh_3_)]} and ^195^Pt {for [Pt(9AA)Cl(DMSO)]} NMR spectroscopies. Molecular and crystal structures for the cyclopalladated [Pd(9AA)(*μ*-Cl)]_2_ ⋅ 2DMF were obtained by X-ray diffraction.

### 3.2 FT-IR

The FT-IR spectra in the range 4000–400cm^−1^ for the 9-aminoacridine and the complexes [Pd(9AA)(*μ*-Cl)]_2_ ⋅ 2DMF, [Pd(9AA)Cl(PPh_3_)], and [Pt(9AA)Cl(DMSO)] were recorded.

In the zone between 1670–1550cm^−1^ the stretching *ν* (> C=N−)^1^ and bending *δ* (NH_2_) bands can be assigned. The *δ* (NH_2_) band of the free ligand appears at 1670 cm^−1^ . In the spectrum of the compound [Pd(9AA)Cl(PPh_3_)], the form of the bands between 550–520cm^−1^, corresponding to the triphenylphosphine molecules [44], confirms the coordination of the palladium atom to only one molecule of PPh_3_ .

In the case of the complex [Pt(9AA)Cl(DMSO)], the IR spectrum shows an additional band at 1032 cm^−1^ assigned to the stretching vibration *ν*(> S=O) of the dimethylsulfoxide molecule coordinated to platinum atom through the sulphur atom [45].

### 3.3 ^1^H NMR spectra

The ^1^H NMR spectra assignments, corresponding to the free ligand and the complexes [Pd(9AA)(*μ*-Cl)]_2_ ⋅ 2DMF, [Pd(9AA)Cl(L)] (L = py- *d*_5_ or PPh_3_) and [Pt(9AA)Cl(DMSO)], are collected in Table 1. All the spectra were recorded in DMSO- *d*_6_ at room temperature with exception of the complex [Pt(9AA)Cl(DMSO)] which was recorded in acetone-*d*_6_. COSY and TOCSY experiments were used for the assignment.

The spectrum of the free ligand shows four signals in the aromatic zone, which indicates chemical equivalence of the protons localized to both sides of the symmetry plane of the molecule. The signals were assigned as described in the literature [46]. The spectra of the complexes are similar: they present seven signals (between 6 and 9 ppm) assigned to the protons of the aromatic CH groups, which demonstrates the loss of symmetry of the ligand as a consequence of the binding to the metal ion. The presence of only seven resonances indicates the formation of a *σ* (Pd–Csp^2^, aryl) bond. On the other side, two singlets assigned to the protons of NH groups appear, confirming the imino form for the ligand.

The value of the chemical shift of proton NH^10^ is *δ* = 11.65 ppm for the palladium complexes and *δ* = 10.73 ppm for the platinum complex. This suggests the presence in solution of a hydrogen bond between the proton bound to endocyclic nitrogen and the DMF molecule present in the three palladium compounds. This type of interaction can also be observed in solid state, in the crystal structure of compound [Pd(9AA)(*μ*-Cl)]_2_ ⋅ 2DMF. In Figure 2 the 2D spectrum for this complex is represented.

The spectra of the monomeric palladium compounds [Pd(9AA)Cl(L)] (L = py- *d*_5_ or PPh_3_) show, as most significant feature, a strong upshift of the H^2^ (see Table 1). This fact is usually observed for the proton in *orto* position relative to metallated carbon atom in cyclopalladate complexes similar to those studied here [47] and it is due to the proximity of this proton to the aromatic ring of pyridine-*d*_5_ or triphenylphosphine in *cis* position relative to the metallated carbon atom.

**Figure 1:**
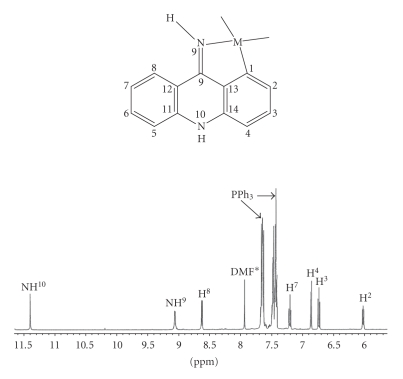
^1^H NMR spectrum (zone 6–11.5ppm) of the complex [Pd(9AA)Cl(PPh_3_)]. *Signal assigned to the DMF present in the precursor complex [Pd(9AA)(*μ*-Cl_2_)] ⋅ 2DMF.

**Figure 2:**
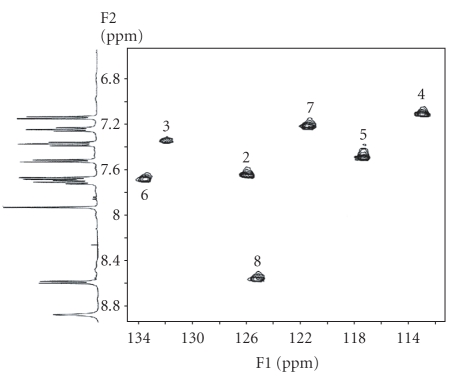
^1^H–^13^C bidimensional heterocorrelated NMR spectrum of the compound [Pd(9AA)(*μ*-Cl)]_2_ (aromatic zone) where seven peaks of crossover are observed as indicator of the presence of a *σ* (M–Csp^2^, aryl) bond.

A noticeable feature in the spectrum of [Pd(9AA)-Cl(PPh_3_)] (see Figure 1) is that the signal of the iminic proton (NH^9^) appears as a doublet due to the coupling with the ^31^P (^3^ *J*_P-H^9^_ = 5 Hz) nucleous. For the same reason, the H^2^ resonance appears as a doublet of doublets (^4^ *J*_P-H^2^_ = 5 Hz). The values of these coupling constants are similar to those of analogue compounds described in the literature [48].

**Figure 3:**
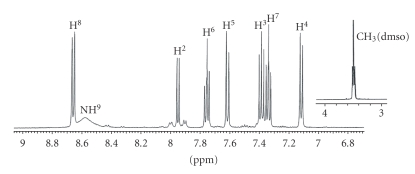
^1^H NMR spectrum of the complex [Pt(9AA)Cl(DMSO)].

In the spectrum of the platinum derivative (see Figure 3), a signal at 3.49 ppm assigned to the protons of the two methyl groups of DMSO appears. The value of the chemical shift as well as the presence of satellites due to the coupling with the ^195^Pt nucleous (^3^ *J*_Pt-H(dmso)_ = 21 Hz) indicate that the DMSO molecule is coordinated to the metal ion. The coupling between the platinum atom and the H^2^ (^3^ *J*_Pt-H^2^_ = 48 Hz) was also identified. On the contrary, the coupling between the iminic proton NH^9^, was not observed, probably due to the width of the signal.

### 3.4 NMR ^13^C{^1^H}

^13^C{^1^H} NMR spectra of the free ligand and the complexes [Pd(9AA)(*μ*-Cl)]_2_ ⋅ 2DMF, [Pd(9AA)Cl(PPh_3_)], and [Pt(9AA)Cl(DMSO)] were obtained.

The signals observed in the ^13^C{^1^H} for the ligand were assigned as previously described in the literature [21]. The spectra of the complexes [Pd(9AA)(*μ*-Cl)]_2_ ⋅ 2DMF, [Pd(9AA)Cl(PPh_3_)] and [Pt(9AA)Cl(DMSO)] show very similar general characteristics. Bidimensional heterocorrelation experiments ^1^H–^13^C were used for the assignments.

The seven crossover single peaks of the aromatic zone in bidimensional experiments (see Figure 3), which are coincidental with the most intense resonances in the ^13^C{^1^H} NMR spectrum, confirm the formation of a *σ* (M–Csp^2^, aryl) (M = Pd or Pt) bond.

^13^C{^1^H} NMR spectra show as well five signals without crossover peaks in herecorrelation study that were assigned to quaternary carbons. The less strong of these resonances that appears downshift to the free ligand was assigned to the metallated carbon (C^1^). This assignment is confirmed by the existence of ^195^Pt satellites in the compound [Pt(9AA)Cl(DMSO)] and for the doublet form due to the coupling with ^31^P nucleous in the complex [Pd(9AA)Cl(PPh_3_)].

The C^9^ signal appears in the range 171–173 ppm, in good agreement with the values described in the literature for iminic carbons in five- or six-member ring cyclometallated compounds derivatives of *Schiff* bases [49].

In the ^13^C{^1^H}NMR spectrum of [Pt(9AA)Cl(DMSO)], the couplings with ^195^Pt nucleous are clearly observed. The values for the coupling constants (Supplementary Material available online at doi 10.1155/2007/98732.) are in good agreement with those described in the literature [50].

### 3.5 ^31^P{^1^H} NMR spectra

The ^31^P{^1^H} spectrum of [Pd(9AA)Cl(PPh_3_)] (in DMSO-*d*_6_) shows a singlet at 40.77 ppm. The position and multiplicity of this signal is consistent with the data described in the literature for the five-membered rings palladacycles with *σ* (Pd–Csp^2^, aryl) bonds [43, 51] of general formulae [Pd(C^N)X(PPh_3_)] (X = Cl^−^, Br^−^, I^−^ or AcO^−^), where the phosphine is in *trans* to the iminic nitrogen.

### 3.6 ^195^Pt NMR spectrum

The complex [Pt(9AA)Cl(DMSO)] has been also characterized by ^195^Pt NMR (in acetone-*d*_6_). The spectrum consists in a singlet at − 3756 ppm. The position of the signal [52] is similar to that described in the literature for mono- or bis-cycloplatinated compounds of formulae [Pt{[(*η* ^5^-C_5_H_3_)CH(R)N (CH_3_)_2_]Fe(*η* ^5^-C_5_H_5_)}Cl (DMSO)] (− 3763 ppm for R = H and − 3899 ppm for R = CH_3_) [50] and [Pt_2_[{*η*5-C_5_H_3_CH(R)N(CH_3_)_2_} Fe]Cl_2_(DMSO)_2_] (R = H or CH_3_) [53] where the platinum(II) is surrounded by a CNSCl coordination environment and the ligand DMSO is in *trans* position to the nitrogen atom bound to the metal ion.

### 3.7 Crystal structure of [Pd(9AA) (*μ*-Cl)]_2_ ⋅ 2DMF

**Figure 4:**
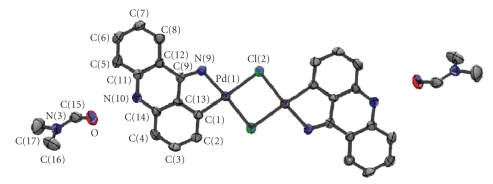
Molecular structure of the complex [Pd(9AA)(*μ*-Cl)]_2_ ⋅ 2DMF.

The main crystallographic data and structure refinement are collected in Table 2. The molecular structure of the complex with the numbering of the atoms is shown in Figure 4. The bond distances and angles are included in Supplementary material. The structure consists in molecules of [Pd(9AA)(*μ*-Cl)]_2_ and DMF in a molar relationship 1:2.

In the [Pd(9AA)(*μ*-Cl)]_2_ molecules, the palladium atoms {Pd(1) and Pd(1^#^)} are bound to two lligands {Cl(2) and Cl(2^#^)} in *cis* position which act as bridges between the two metal centres. The two coordination sites are occupied by the *exocyclic* nitrogen {N(9)} and the carbon atom C(1) from the 9-aminoacridine ligand, confirming the proposal of the formation of a *σ* (Pd–Csp^2^, aryl) bond, and the bidentate and monoanionic ligand condition of the 9-aminoacridine.

The molecule could be viewed as the combination of fragments “[Pd(9AA)(*μ*-Cl)]” sharing chlorine bridges to form a “Pd(*μ*-Cl)_2_ Pd” unit. The relative position of the metalated ligands is in *trans,* and the environment of palladium atom is a slightly distorted square plannar.^1^

The bond distance Pd-N {2.023(3)Å} is similar to those described in the literature for five-membered ring palladacycles derivatives of organic ligands with > C=N− groups, such as imines [54], oximes [55], or hydrazones [56]. The bond length for (Pd–C) {1.987(2)Å} is in good agreement with those expected for the bonds between palladium(II) and a Csp^2^, aryl (these bond distances are typically in the range 1.98Å and 2.10Å) [43, 56–60].

The bond angles involving palladium atom between 82.72(11)° {C(1)-Pd(1)-N(9)} and 94.31(9)° {Cl(2)-Pd(1)-Cl(2^#^)} are in good agreement with those described in the literature for five-membered ring cyclopalladated dinuclear complexes [Pd(C^N)(*μ*-X)]_2_ .

Each of the two halves of the molecule has a five-membered metallocycle formed by Pd(1), N(9), C(9), C(12), and C(13) atoms, which is practically planar ^2^ and forms a 3.3° angle with the coordination plane of the metal.

The units “Pd(*μ*-Cl)_2_ Pd” are asymmetric, as indicated by the value of the bond distances Pd(1)–Cl(2): 2.4741(13) Å and Pd(1)–Cl(2^#^): 2.352(2) Å. This result is consistent with the structural data obtained for dimeric cyclopalladated complexes with chlorine bridges and *trans* configuration of the two palladate groups of general formula [Pd(C^N)(*μ*-Cl)]_2_. This is a consequence of the different influence of the atoms in *trans* position {the metallated carbon (C1) and the nitrogen N(9)} [43].

**Figure 5:**
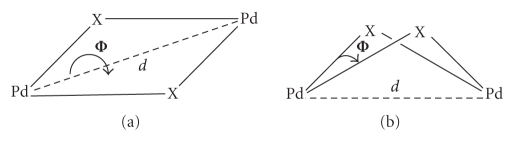
Geometry of the fragment “Pd(*μ*-X)_2_ Pd” and dependence of the distance Pd ⋯ Pd with the angle Φ.

The planar unit “Pd(*μ*-Cl)_2_ Pd”^3^ forms a 4.9° angle with the metallocycle (see Figure 5) and it has a rhomboidal shape. The distance between the two palladium atoms, 3.479(1)Å, is too large to consider the existence of significant Pd ⋯ Pd interactions.

The bond angle Cl(2)–Pd(1)–Cl(2^#^) is similar to those described for compounds of general formula *trans*-[Pd(C^N)(*μ*-Cl)]_2_ containing five-membered metallocycles [61].

the tricyclic system [6,6,6] of the aromatic ligand is practically planar ^4^ and it forms a 2.4° angle with the palladacycle. This is in contrast with what occurs in other reported structures [22] where the 9-aminoacridine is bound to the metal through its exocyclic nitrogen is also present as imino tautomer and a folding of the side rings with respect to the N(10)–C(9) vector was found. One of the most significant structural features of the 9-aminoacridine group in the compound [Pd(9AA)(*μ*-Cl)]_2_ is the bond length N(9)–C(9), 1.312(3)Å, which is appreciably smaller than the expected value for a *σ* (Csp^2^–Nsp^3^) bond and very similar to those described in the literature for complexes containing the functional group >C=N− (see footnote 1) and also for the platinum compound [Pt(L)Cl], where L = 1-nitro-9-[{2-(dimethylamino)ethyl}amino]acridine [22] with the presence of the tautomeric form imino.

Moreover, the bond distances N(10)–C(11) 1.399(4) Å and N(10)–C(14) {1.379(3) Å} and the bond angle C(11)–N(10)–C(14) {122.3(2)°} in the complex [Pd(9AA)(*μ*-Cl)]_2_ are higher than expected for an amino form and very similar to those found for the cited platinum complex [22]. These observations allow to conclude that in the complex [Pd(9AA)(*μ*-Cl)]_2_ the ligand is present in the imino form and that the functional group >C=N− is included in the five-membered metallocycle.

The bond distances and angles found for the two molecules of dimethylformamide present in the crystal structure of [Pd(9AA)(*μ*-Cl)]_2_ ⋅ 2DMF are similar to those described in the literature for complexes containing DMF crystallization molecules. The distance between the oxygen atom of DMF and the hydrogen bound to the *endocyclic* nitrogen, N(10), of the complex [Pd(9AA)(*μ*-Cl)]_2_ {O ⋯ H–N(10)= 2.857(4)Å} suggests the existence of intermolecular hydrogen interactions. The strong downfield shift observed in the proton NMR spectrum assigned to the H^10^ (*δ* = 11.65 ppm), could confirm the existence of these interactions in solution.

## 4. BIOLOGICAL STUDIES

### 4.1 Circular Dichroism

**Figure 6:**
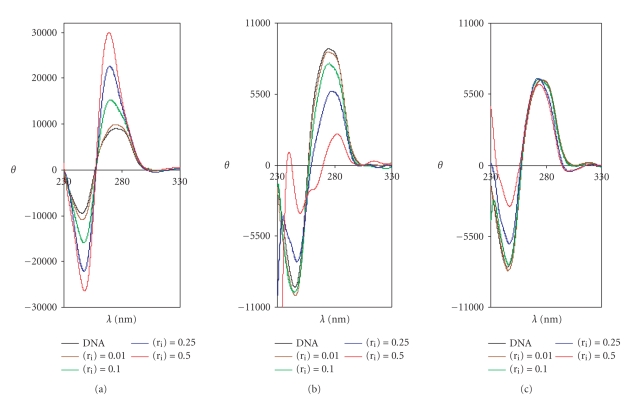
DC spectra of *Calf Thymus* DNA incubated with (a) 9-aminoacridine (9AA), (b) [Pd(9AA)(*μ*-Cl)]_2_, and (c) [Pt(9AA)Cl(DMSO)].

The molecules of 9-aminoacridine (9AA) and complexes [Pd(9AA)(*μ*-Cl)]_2_ and [Pt(9AA)Cl(DMSO)] originate modifications in the spectrum of *Calf Thymus* DNA at different values of (r_i_) selected as it is shown in Figure 6. The wavelengths, corresponding to the maximum and minimum values of the ellipticity, are collected in Table 3.

The complexes [Pd(9AA)(*μ*-Cl)]_2_ and [Pt(9AA)Cl-(DMSO)] can interact with the DNA, in principle, by intercalation of the ligand and/or forming a covalent bond through the metal ion.

Looking at the spectrum recorded for the CT-DNA incubated with the complex [Pd(9AA)(*μ*-Cl)]_2_ (see Figure 6(b)), a very strong decreasing of the ellipticity of both, the positive and negative bands, and a *batochromic* shift of the bands can be observed. This is the opposite effect to that observed for the free 9-aminoacridine (9AA) (see Figure 6(a)) . In the case of the platinum compound [Pt(9AA)Cl(DMSO)] (see Figure 6(c)) , the change in the negative band is similar to that of the palladium compound but the maximum is much less intense.

On the other hand, the amounts of metal incorporated to the DNA are much higher for the palladium compound than for the platinum compound (see Table 3 ). Moreover, the complex [Pd(9AA)(*μ*-Cl)]_2_ gives a percentage of metal incorporation higher than *cisplatin* at the molar relationships (r_i_) used. The small percentage of platinum incorporated to DNA for [Pt(9AA)Cl(DMSO)] may be conditioned by the different labilization kinetic of 9-aminoacridine and chlorine ions and/or the fact that the compound [Pt(9AA)Cl(DMSO)] contains only a hydrolysable chlorine and the most probable adduct that could be formed with DNA would be monofunctional.

In conclusion, the 9-aminoacridine behaves as a classical intercalator but the palladium and platinum complexes produce changes of different nature, probably due to the formation of a covalent bond or the occurrence of both interactions, covalent, and intercalation, simultaneously.

### 4.2. Electrophoretic mobility

**Figure 7:**
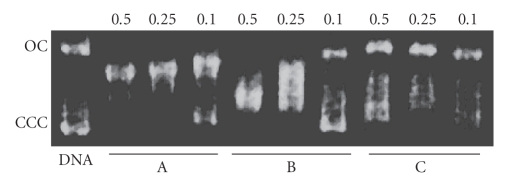
Eletrophoretic mobility pattern of pBR322 plasmid DNA incubated with the complexes: lane A: cisplatin; lane B: [Pd(9AA)(*μ*-Cl)]_2_; lane C: 9AA.

In Figure 7, the electrophoretic mobility pattern of cisplatin, 9-aminoacridine and the complex [Pd(9AA)(*μ*-Cl)]_2_ are shown. At low values of (r_i_), a decreasing of the CCC form mobility for the free 9-aminoacridine molecule (lane C) can be observed. However, at (r_i_) = 0.5, the mobility increases. This behavior is similar to that observed for cisplatin at higher concentrations than the ones used in this study. The intercalation interaction usually causes a higher degree of supercoiling than the one produced by a covalent cis-bifunctional binding [62].

On the other hand, the electrophoretic behavior of pBR322, incubated with the compound [Pd(9AA)(*μ*-Cl)]_2_ (Figure 7, lane B), is close to that of cisplatin (Figure 7, lane A) which seems to indicate that the interaction of this compound is not only intercalative. This seems to agree with the results obtained from the circular dicroism study described in the previous section.

**Figure 8:**
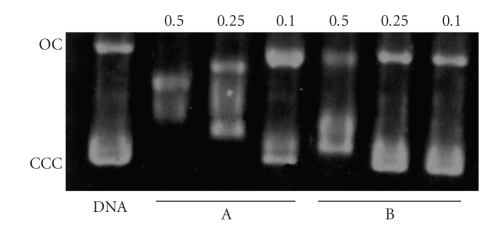
Electrophoretic mobility pattern of pBR322 plasmid DNA incubated with the complexes: lane A: cisplatin; lane B: [Pt(9AA)Cl(DMSO)].

Finally, the complex [Pt(9AA)Cl(DMSO)] (Figure 8, lane B) causes slight modifications in the electrophoretic mobility of the OC while the CCC form retards, which suggests that the uncoiling of the helix occurs on a minor degree. It is possible that, in addition to the intercalation, a monofunctional covalent binding could be established. This result would agree with the published results for the complex [PtCl(dien)]Cl, which uncoils the helix about 6°, half of the value expected for a cis bifunctional binding as int the values described for cisplatin [62].

### 4.3. Atomic force microscopy

**Figure 9:**
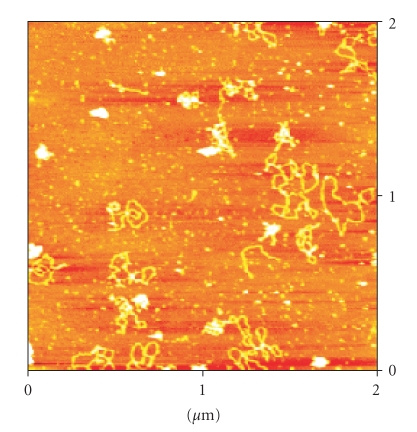
AFM image of the pBR322 plasmid DNA incubated with the complex [Pd(9AA)(*μ*-Cl)]_2_.

**Figure 10:**
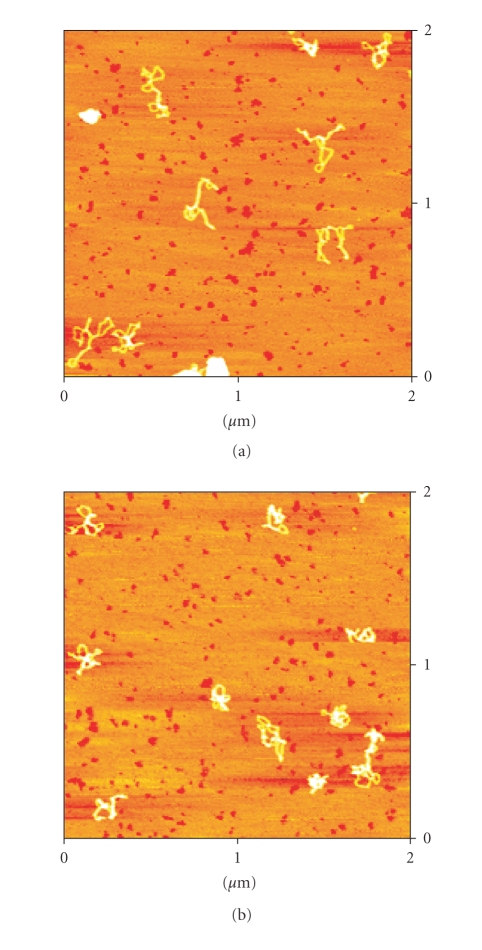
Two AFM images corresponding to pBR322 plasmid DNA incubated with the complex [Pd(9AA)Cl(PPh_3_)].

AFM images of the plasmid pBR322 DNA incubated with the compounds [Pd(9AA)(*μ*-Cl)]_2_ and [Pd(9AA)Cl(PPh_3_)] for 5 hours and 37°C are presented in Figures 9 and 10, respectively.

In all the images supercoiled forms of the plasmid DNA can be observed. These modifications are likely to correspond to the strong effect of intercalation of the 9- aminoacridine ligand.

Supercoiling in the plasmid DNA tertiary structure has been observed before for other classic intercalators such as ethydium bromide and planar heterocycles [63, 64].

In the case of the complex [Pd(9AA)Cl(PPh_3_)], additional interaction, probably due to the formation of covalent bond with the N atom of the purine bases, originates deeper changes in the structure of the plasmid

### 4.4. Antiproliferative assays

The “in vitro” growth inhibitory effect of the 9-aminoacridine and its palladium complex [Pd(9AA)(*μ*-Cl)]_2_ were evaluated in three tumor cell lines: MCF-7 breast cancer cell line, DU-145 prostate cancer cell line, and HeLa cervix cancer cell line.

In Table 4, the IC_50_ values for the two compounds against the three tumor cell lines are collected. The 9-aminoacridine again presents low IC_50_ values against the tumor cell lines assayed. These results suggest a direct correlation with the conclusions drawn from the studies of interaction with DNA in previous sections. Many other compounds related to acridines have demonstrated intercalation in DNA and antiproliferative behavior [65]. Although the [Pd(9AA)(*μ*-Cl)]_2_ derivative shows higher IC_50_ values than cisplatin, the parameters are low enough to merit consideration in further biochemical studies.

## Figures and Tables

**Table 1:** ^1^H chemical shifts (ppm) of 9-aminoacridine (9AA) and the complexes [Pd(9AA)(*μ*-Cl)]_2_ ⋅ 2DMF, [Pd(9AA)Cl(L)] (L = py-*d*_5_ or PPh_3_) and [Pt(9AA)Cl(DMSO)]. The numbering corresponds to the attached scheme and it will be the same along the spectroscopic studies.

	9AA	[Pd(9AA)(μCl)]^&^_2_	[Pd(9AA)Cl(py-*d*_5_)]^&, #^	[Pd(9AA)Cl(PPh_3_)]^&^	[Pt(9AA)Cl(DMSO)]^$^
H^1^	8.40 d	—	—	—	—
H^2^	7.65 t	7.71 d	5.89 d	6.04 dd	7.95 d
H^3^	7.32 t	7.37 t	7.19 t	6.75 t	7.39 t
H^4^	7.80 d	7.14 d	7.11 d	6.88 d	7.12 d
H^5^	7.80 d	7.53 d	7.59 d	7.48	7.62 d
H^6^	7.32 t	7.74 t	7.71 t	7.62	7.76 t
H^7^	7.65 t	7.23 t	7.25 t	7.22 t	7.34 t
H^8^	8.40 d	8.66 d	8.60 d	8.64 d	8.66 d
NH^9^	—	8.94 s	8.78 w	9.08 d	8.58 w
NH^10^	—	11.65 s	11.68 w	11.42 s	10.73 w

s: singlet, d: doublet, t: triplet, w: wide, dd: doublet of doublets.

^&^DMF signals were assigned at 7.99, 2.89 and 2.30 ppm.

^#^Pyridine- *d*_5_ signals were assigned at 7.40 and 7.80 ppm.

^$^Recorded in acetone- *d*_6_. The spectrum shows an additional singlet at 3.49 ppm assigned to the protons of the CH_3_ groups of DMSO (^3^ *J*_Pt-H(DMSO)_ = 21 Hz).

**Table 2:** Crystal data and structure refinement for [Pd(9AA)(*μ*-Cl)]_2_ ⋅ 2DMF.

Empirical formula	C_26_H_30_Cl_2_N_8_Pd_2_
Formula weight	738.28
Crystal size (mm)	0.1 × 0.1 × 0.2
Crystal system	Monoclinic
Space group	P2_1_ /c
a, b, c (′)	a = 12.421(6) b = 9.538(5) c = 14.476(14)
*α*, *β*, *γ* (°)	*α* = *γ* = 90.0 *β* = 109.07(5)
Volume (Å^3^)	1620.9(19)
Z	1
Density (calc.) (Mg × m^−3^)	0.756
Absorption coefficient (mm^−1^)	0.651
F(000)	368
Temperature (K)	293(2)
Wavelength (′)	0.71069
*θ* range (°)	2.60–29.97
Reflexions collected	4911
Unique reflexions	4715 [R(int) = 0.0182]
Data/restraints/parameters	4665/0/200
Goodness-of-fit on F^2^	1.007
Final R	R1 = 0.0336, wR1 = 0.0895
R (all data)	R1 = 0.0507, wR2 = 0.0968

**Table 3:** Ellipticity values and wavelenghts (maximum and minimum) in CD spectra of *Calf Thymus* DNA incubated with 9-aminoacridine (9AA) and its palladium and platinum complexes.

Compound	r_i_	*θ* ^(a)^_max_	*λ* ^(b)^_max_	*θ* ^(a)^_min_	*λ* ^(b)^_min_	% uptaken metal
DNA^(c)^	—	9.0	275.0	−9.5	245.5	—

9AA	0.01	9.8	274.1	−10.9	246.0	—

	0.10	15.3	270.0	−15.9	247.0	—
	0.25	22.7	269.2	−22.1	247.0	—
	0.50	30.0	268.8	−26.5	247.8	—

[Pd(9AA)(*μ* −Cl)]_2_	0.01	8.8	274.5	−10.1	245.8	62.24

	0.10	7.8	274.4	−9.8	244.8	51.82
	0.25	5.7	277.6	−7.4	247.0	50.48
	0.50	2.4	282.4	−3.7	250.0	52.67

DNA^(d)^	—	6.6	276.0	−7.9	245.0	—

[Pt(9AA)Cl(DMSO)]	0.01	6.6	276.0	−8.1	245.6	9.72

	0.10	6.7	272.8	−7.7	245.8	7.43
	0.25	6.7	272.5	−6.0	246.5	6.21
	0.50	6.2	273.8	−	247.1	5.44

^(a)^degrees × cm^2^ × dmol^−1^ × 10^3^;

^(b)^nm;

^(c)^6.49 × 10^−5^ mol × l^−1^;

^(d)^6.10 ×10^−5^ mol × l^−1^.

**Table 4:** IC_50_ values (*μ*M) for the compounds studied against the tumor cell lines MCF-7, DU-145, and HeLa.

Compound	MCF-7	DU-145	HeLa
cisplatin	9.4	3.7	22.2
9AA	4.9	11.9	22.2
[Pd(9AA)(*μ*-Cl)]_2_	38.1	32.6	>50

## Footnotes

The equation of the plane defined by the atoms Cl(2), Cl(2^#^), N(9), and C(1) is (−0.1236)XO + (0.9015)YO + (0.4146)ZO = 4.2986. The deviations related to the main plane are Cl(2): −0.035, Cl(2^#^): 0.037, N(9): 0.045, and C(1): −0.047 Å.

The equation of the plane defined by the atoms Pd(1), N(9), C(9), C(1), and C(13) is (−0.1700)XO + (0.9087)YO + (0.3808)ZO = 4.2078. The deviations related to the main plane are Pd(1): −0.019, N(9): −0.023, C(9): 0.015, C(1): −0.019, and C(13): 0.008Å.

The equation of the plane defined by the four atoms involved in the unit “Pd(*μ*-Cl) Pd” is (−0.1050)XO + (0.8943)YO + (0.4350)ZO = 4.2649.

The equation of the plane defined by the 14 atoms involved in the tricycle system of the acridine molecule is (−0.2109)XO + (0.9051)YO + (0.3693)ZO = 4.0107.
